# Five lipoxygenase hypomethylation mediates the homocysteine effect on Alzheimer’s phenotype

**DOI:** 10.1038/srep46002

**Published:** 2017-04-06

**Authors:** Jian-Guo Li, Carlos Barrero, Salim Merali, Domenico Praticò

**Affiliations:** 1 Department of Pharmacology and Center for Translational Medicine, Lewis Katz School of Medicine, Temple University Philadelphia, PA 19140, USA; 2 Department of Pharmaceutical Sciences, Temple University Philadelphia, PA 19140, USA.

## Abstract

Environmental and genetic risk factors are implicated in the pathogenesis of Alzheimer’s disease (AD). However, how they interact and influence its pathogenesis remains to be investigated. High level of homocysteine (Hcy) is an AD risk factor and associates with an up-regulation of the ALOX5 gene. In the current paper we investigated whether this activation is responsible for the Hcy effect on the AD phenotype and the mechanisms involved. Triple transgenic mice were randomized to receive regular chow diet, a diet deficient in folate and B vitamins (Diet), which results in high Hcy, or the Diet plus zileuton, a specific ALOX5 inhibitor, for 7 months. Compared with controls, Diet-fed mice had a significant increase in Hcy levels, memory and learning deficits, up-regulation of the ALOX5 pathway, increased Aβ levels, tau phosphorylation, and synaptic pathology, which were absent in mice treated with zileuton. *In vivo* and *vitro* studies demonstrated that the mechanism responsible was the hypomethylation of the ALOX5 promoter. Our findings demonstrate that the up-regulation of the ALOX5 is responsible for the Hcy-dependent worsening of the AD phenotype in a relevant mouse model of the disease. The discovery of this previously unknown cross-talk between these two pathways could afford novel therapeutic opportunities for treating or halting AD.

Alzheimer’s disease (AD) is the most common form of chronic neurodegenerative condition with dementia in the elderly, affecting approximately 6–8% all persons aged >65 years^1^. While only a minority of AD cases is secondary to missense mutations in genes involved for either Aβ precursor protein (APP), or Presenil-1 and −2, the cause of sporadic AD remains unclear, and a combination of environmental and genetic risk factors has been implicated^2^.

Among them, epidemiological and clinical studies have indicated that elevated circulating level of homocysteine (Hcy), also known as hyper-homocysteinemia (HHcy), is a modifiable risk factor for AD onset^3^,^4^. Hcy is a sulfur-containing amino acid and intermediate product of the methionine cycle, whose normal levels in the body are kept by its re-methylation to methionine in a reaction that requires the availability of folate, vitamin B6 and 12^5^. A diet with excessive methionine, or with significant deficit in folate, B vitamins, or genetic alterations in certain enzymes of the methionine cycle are all conditions known to increase Hcy levels *in vivo*^6^,^7^.

The elucidation of the molecular mechanism underlying the biological effect of HHcy on the development of AD phenotype and pathogenesis is very important since it could provide new insights for the treatment and or prevention of the disease in individuals bearing this risk factor.

Previous studies showed that genetic and diet-induced high Hcy results in significant increase in Aβ levels and deposition in APP transgenic mice^8^,^9^,^10^. More recently, we demonstrated that diet-induced HHcy in the triple transgenic mice (3xTg) results in an exacerbation of behavioral deficits, brain amyloidosis and tau neuropathology^11^. Interestingly, we observed that under this experimental condition mice had a significant up-regulation of the 5Lipoxygenase (5LO) enzymatic pathway^12^. Since 5LO is known to be up-regulated in brain tissues of AD patients^13^, where it plays an active role as an endogenous modulator of Aβ formation and tau phosphorylation^14^,^15^, it remains to be investigated whether the 5LO up-regulation during HHcy is directly involved and essential in modulating the development of AD phenotype secondary to this condition, or it is simply an associated and secondary event to the condition.

To address this important biological question, we tested the hypothesis that diet-induced HHcy-dependent exacerbation of the AD phenotype in 3xTg mice will be rescued by pharmacological inhibition of 5LO activity.

## Results

### *In vivo* study

#### Folate and B vitamins deficient diet-induced behavioral impairments are rescued by zileuton

**Figure 1:**
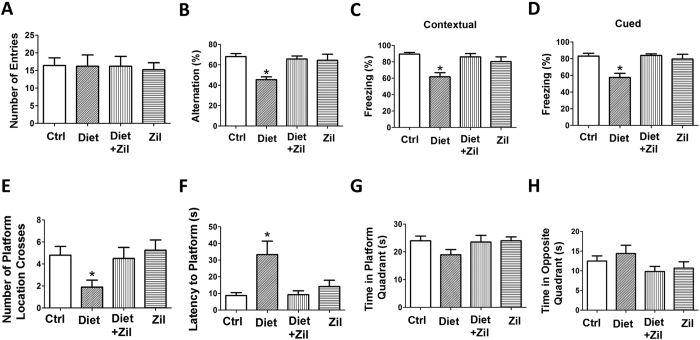
Rescue of diet-induced high Hcy-dependent behavioral deficits by zileuton. (**A**) Number of total arm entries for 3xTg mice receiving regular diet (Ctrl), folate and B vitamin deficient diet (Diet), Diet and zileuton (Diet + Zil), or zileuton alone (Zil). (**B**) Percentage of alternations of the 3xTg mice described in panel A (*p < 0.05). (**C**) Contexual fear memory responses in the four groups of 3xTg mice described in panel A. (**D**) Cued fear memory response in the same 3xTg mice (*p < 0.05). (**E**) Number of entries to the target platform zone for the 3xTg mice described in panel A. (**F**) Latency time to reach the target platform zone for the same four groups of 3xTg mice (*p < 0.05). (**G**) Time spent in the platform quadrant for the same groups of 3xTg mice (*p < 0.05). (**H**) Time spent in the opposite quadrant zoned for the same four two groups of 3xTg mice. Values represent mean ± s.e.m. (n = 5 control, n = 6 Diet, n = 6 Diet + zileuton, n = 5 zileuton).

To investigate the effect of a folate and B vitamins-deficient diet (Diet) on cognition with and without the pharmacological inhibition of 5LO, 3xTg mice were assessed in three different paradigms. In the Y-maze test, we found that all mice in the different groups had a similar total number of arm entries (Fig. 1A). By contrast, compared with controls Diet-treated 3xTg mice had a significant reduction in the percentage of alternation, which was restored in mice that together with the diet received zileuton (Fig. 1B). In the fear conditioning test, compared with controls, 3xTg mice receiving the Diet had a significant lower freezing time in the contextual and cued recall paradigms, which was absent in mice receiving the Diet plus zileuton (Fig. 1C,D). Finally, we examined the effect of the Diet on the reference spatial memory function of the mice by using the Morris water maze. In the study, we performed a visible platform cued test followed by hidden platform testing with four training trials per day. Mice in all groups were able to reach the training criterion within 4 days and were similarly proficient swimmers (data not shown). However, compared with controls, in the probe test we found that Diet-treated 3xTg mice had a significant decrease in the number of platform location crosses (Fig. 1E), and an increase in the latency to reach the platform (Fig. 1F), but no statistical differences were note for the time spent in the platform quadrant (Fig. 1G) and the opposite quadrant (Fig. 1H). By contrast, mice that together with the Diet received zileuton were indistinguishable from controls in the number of crosses and latency to platform (Fig. 1E–H). In all of these tests zileuton alone was without any significant effect (Fig. 1A–H).

#### Folate and B vitamins deficient diet results in high brain homocysteine and up-regulation of the 5LO enzymatic pathway

Confirming compliance of the animals with the chronic folate and B vitamins deficient dietary regimen, brain Hcy levels in these mice were significant higher than the control group, but unaffected by the administration of zileuton (Table 2).

**Figure 2:**
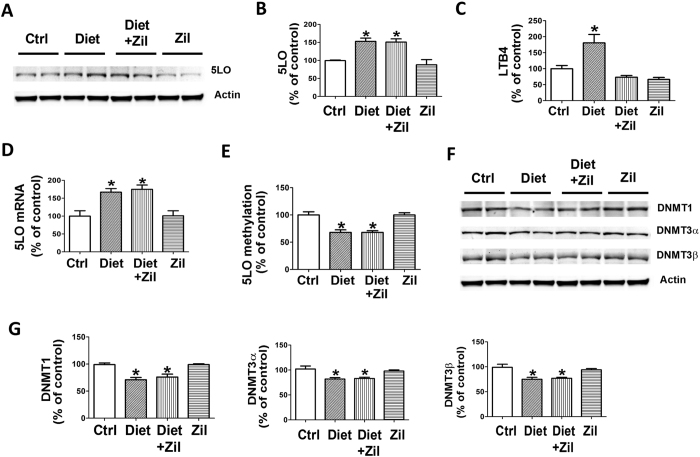
Diet-induced high Hcy upregulates 5LO enzymatic pathway via epigenetic modification. (**A**) Representative western blot analysis of 5LO protein in brain cortex homogenates from 3xTg mice receiving vehicle (Ctrl), folate and B vitamins deficient diet (Diet), Diet + zileuton, or zileuton. (**B**) Densitometric analysis of the immunoreactivity to the antibody shown in panel A. (**C**) Levels of LTB4 measured by a specific and sensitive ELISA assay in brain cortex homogenates from the same four groups of 3xTg mice. (**D**) Quantitative real time Reverse Transcription Polymerase Chain Reaction (q RT-PCR) analysis of 5LO mRNA in brain cortices of 3xTg mice receiving vehicle (Ctrl), folate and B vitamins deficient diet (Diet), Diet + zileuton, or zileuton. (**E**) 5LO DNA methylation levels in brain cortices of the 3xTg mice randomized to the same four treatment groups. (**F**) Representative western blot analyses for DNMT1, DNMT3α, DNMT3β in brain cortices of the same four groups of mice. (**G**) Densitometric analyses of the immuno-reactivity shown in the previous panel. Data presented are mean ± s.e.m. (*p < 0.05, n = 6). (n = 5 control, n = 6 Diet, n = 6 Diet + zileuton, n = 5 zileuton).

Compared with controls, brain homogenates from mice receiving the Diet had a significant increase in the steady state levels of 5LO protein, mRNA and activity as shown by the significant elevation of its main metabolic product, the leukotriene (LT)B4 (Fig. 2A–D). However, while the 5LO protein and mRNA levels were unaffected by zileuton, as expected the drug at the concentration used, which was based on our previous work^16^, prevented the Hcy-dependent LTB4 increase (Fig. 2A–D). Consistent with the elevation in Hcy, the same samples had higher levels of S-adenosyl-homocysteine (SAH), a potent inhibitor of methyl-transferase reactions, and lower S-adenosyl-methionine (SAM) (Table 1). These changes were associated with a significant reduction in all 3 major DNA methyl-transferase enzymes, DNMT1, DNMT3α, DNMT3β, and a significant reduction in 5LO DNA methylation (Fig. 2E–G).

#### Homocysteine-dependent increase in Aβ is prevented by 5LO pharmacological blockade

**Figure 3:**
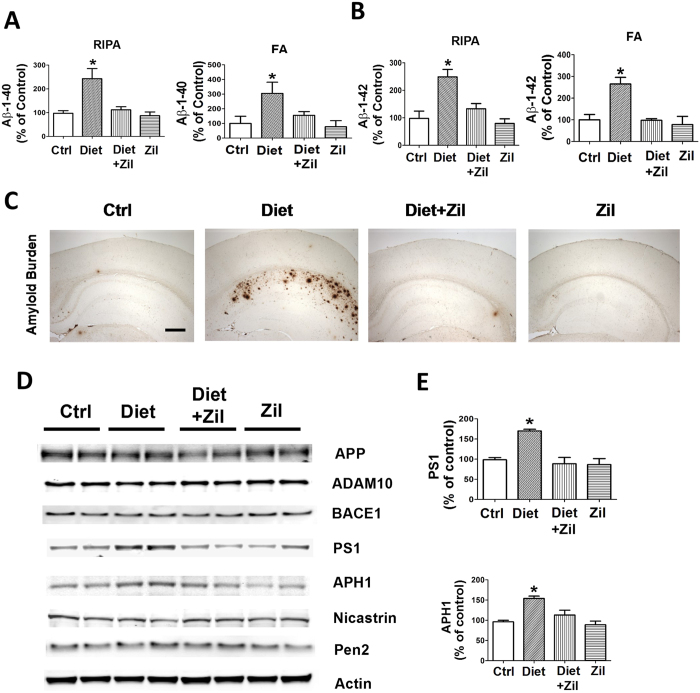
High Hcy-dependent increase in Aβ peptides levels and deposition is prevented by pharmacological blockade of 5LO. (**A,B**) RIPA-soluble (RIPA) and formic acid extractable (FA) Aβ1-40 and Aβ1-42 levels in brain cortex of 3xTg receiving vehicle (Ctrl), folate and B vitamin deficient diet (Diet), Diet + zileuton, or zileuton measured by sandwich ELISA (*p < 0.05). (**C**) Representative images of brains from the four groups of 3xTg mice immunostained with 4G8 antibody. (Scale bar: 500 μm). (**D**) Representative western blots of APP, ADAM-10, BACE-1, PS1, APH-1, Nicastrin, Pen-2, in brain cortex homogenates from 3xTg mice receiving vehicle (Ctrl), the diet (Diet), Diet plus zileuton, or zileuton. (**E**) Densitometric analyses of the immunoreactivities to the antibodies shown in the previous panel (*p < 0.05). Values represent mean ± s.e.m. (n = 5 control, n = 6 Diet, n = 6 Diet + zileuton, n = 5 zileuton).

Next, we investigated the effect of Diet-induced high Hcy on brain Aβ levels and deposition in the 3xTg in the presence or absence of zileuton. As shown in Fig. 3A,B, we found that mice with elevated brain Hcy had a significant increase in both soluble (RIPA-extractable) and insoluble (Formic acid-extractable) Aβ1-40 and Aβ1-42 levels when compared with the control group. Brain Aβ deposition studies by immunohistochemistry confirmed this observation, showing that the Aβ immuno-positive areas occupied by 4G8 immuno-reactions were significantly higher in the Diet-treated mice than in the control group (Fig. 3C). By contrast, mice that together with the Diet were treated with zileuton did not manifest any significant changes in Aβ levels and deposition when compared with control (Fig. 3A–C). Zileuton alone had no significant effect on Aβ levels and deposition (Fig. 3A–C).

Western blot analyses showed that Diet-treated mice had a significant increase in the steady state levels of two of the four components of the γ-secretase complex, PS1 and APH-1, which were absent in the Diet group treated with zileuton (Fig. 3D,E). On the other hand, no changes in the steady state levels of APP, α-secretase (ADAM-10), β-secretase (BACE-1) were observed in any of the different group (Fig. 3D,E). Zileuton alone had no effect on any of these proteins (Fig. 3D,E).

#### Homocysteine-induced increase in tau phosphorylation is prevented by zileuton

**Figure 4:**
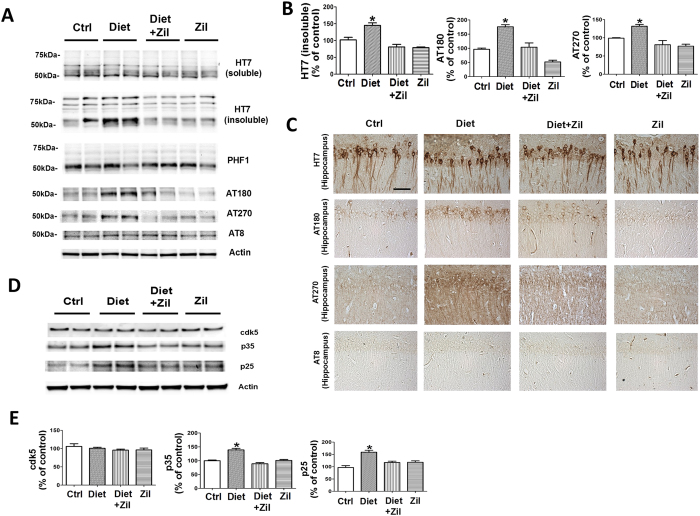
Diet-induced high Hcy-dependent increase in tau phosphorylation is blocked by zileuton. (**A**) Representative western blots of soluble and insoluble total tau (HT7), phosphorylated tau at residues S396/S404 (PHF-1), T231/S235 (AT180), at T181 (AT270), and S202/T205 (AT8), in brain cortex homogenates from 3xTg mice receiving vehicle (Ctrl), folate and B vitamins deficient diet (Diet), Diet + zileuton, or zileuton. (**B**) Densitometric analyses of the immunoreactivities to the antibodies shown in the previous panel (*p < 0.05). (**C**) Representative images of brain sections from the same four groups of 3xTg mice immunostained with HT7, AT8, AT180, AT270 antibodies (Scale bar: 100 μm). (**D**) Representative western blots of cdk5, p35, and p25 in brain cortex homogenates from 3xTg mice treated with vehicle (Ctrl), supplemented diet (Diet), Diet + zileuton, or zileuton. (**E**) Densitometric analyses of the immunoreactivities to the antibodies shown in the previous panel (*p < 0.05). Values represent mean ± sem. (n = 5 control, n = 6 Diet, n = 6 Diet + zileuton, n = 5 zileuton).

Compared with controls, Diet-treated mice had a significant increase in the insoluble tau fraction while there was no change in the level of total soluble tau (Fig. 4A,B). In addition, we found that brains form the same mice had higher levels of tau phosphorylation at specific epitopes: at T231/S235, as recognized by the antibody AT180, and at T181, as recognized by the antibody AT270, but no changes at S396, as recognized by the antibody PHF-13; S396/404, as recognized by the antibody PHF-1; and S202/T205, as recognized by the antibody AT8 (Fig. 4A,B). By contrast, no changes for any of these parameters were observed in the Diet-treated mice receiving zileuton, or zileuton alone when compared with the control group (Fig. 4A,B).

Consistent with these results, immunohistochemical staining showed increased dendritic accumulations of the same tau phosphorylated isoforms in the brains of the Diet-treated mice, which were absent in the ones that together with Diet were treated with zileuton (Fig. 4C).

As shown in Fig. 4D, we observed that compared with controls, brains of Diet-treated 3xTg mice while did not manifest any changes in the steady state protein levels of the cdk5, had a significant increase in both the p25 and p35 fragments and activators of this kinase, which was prevented by the addition of zileuton to the Diet. The drug alone had no effect on the cdk5 pathway (Fig. 4D,E).

#### The effect of high homocysteine on synaptic integrity and neuroinflammation is reversed by zileuton

**Figure 5:**
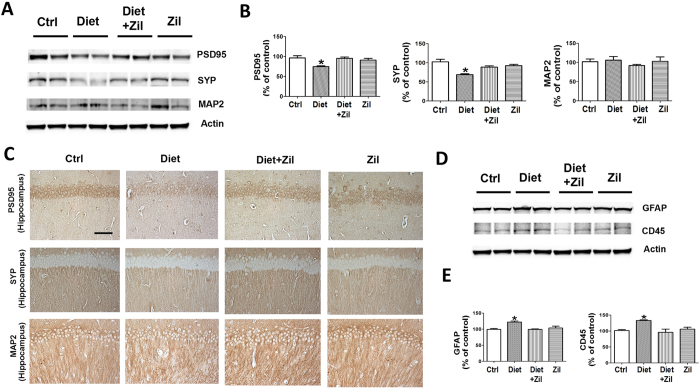
Diet-induced high Hcy brain level affects synaptic integrity and neuroinflammation. (**A**) Representative western blot analyses of post-synaptic density protein 95 (PSD-95), synaptophysin (SYP), and microtubule-associated protein 2 (MAP2) in brain cortex homogenates of controls (ctrl), folate and B vitamins deficient diet (Diet), Diet + zileuton, or zileuton-treated 3xTg mice. (**B**) Densitometric analyses of the immunoreactivities to the antibodies shown in the previous panel (*p < 0.05). (**C**) Representative images of brain sections from 3xTg mice receiving vehicle (Ctrl), supplemented diet (Diet), Diet + zileuton, or zileuton alone immunostained with PDS-95, synaptophysin, and MAP2 antibodies (Scale bar: 100 μm) (**D**) Representative western blots of GFAP and CD45 in brain cortex homogenates from controls (Ctrl), Diet, Diet + zileuton, or zileuton-treated 3xTg mice. (**E**) Densitometric analyses of the immunoreactivities to the antibodies shown in the previous panel (*p < 0.05). Values represent mean ± s.e.m. (n = 5 control, n = 6 Diet, n = 6 Diet + zileuton, n = 5 zileuton).

Next, we investigated the effect of the Diet with and without zileuton on synaptic integrity markers. Compared with controls, steady state levels of two distinct synaptic proteins, synaptophysin and post-synaptic protein-95 (PDS-95), but not MAP-2, were significantly decreased in the mice administered the Diet (Fig. 5A,B). By contrast, mice on the same Diet but simultaneously treated with zileuton had levels of these proteins no different from controls (Fig. 5A,B). Immunohistochemical staining results were consistent with these observations (Fig. 5C). In addition, we observed that compared with controls Diet-treated mice had a significant increase in GFAP and CD45 immunoreactivities, which was blunted in mice that with the Diet also received zileuton (Fig. 5D,E). Zileuton alone had no significant effect on both synaptic integrity and neuroinflammatory markers (Fig. 5A–E).

### *In vitro* study

#### Homocysteine effect on Aβ is mediated by 5LO DNA hypomethylation

**Figure 6:**
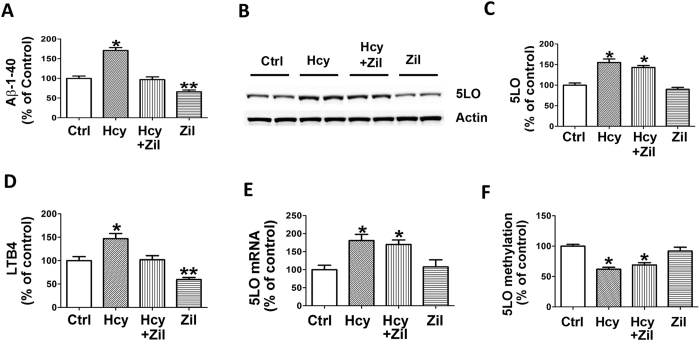
Hcy up-regulates 5LO enzymatic pathway by reducing its DNA methylation. (**A**) Levels of Aβ 1-40 in conditioned media from N2A APPswe cells incubated with vehicle (Ctrl), 50 μM Hcy, Hcy plus zileuton(100 μM), or zileuton (100 μM) alone for 24 hrs. (**B**) Representative western blot analyses of 5LO in lysates from the same N2A APPswe cells described in the previous panel. (**C**) Densitometric analysis of the immune-reactivity shown in panel B. (**D**) Levels of LTB4 measured by a specific ELISA assay in conditioned media from the same N2A APP cells described in panel A. (**E**) Quantitative real time Reverse Transcription Polymerase Chain Reaction (q RT-PCR) analysis of 5LO mRNA in N2A APPswe neuronal cells treated with vehicle (Ctrl), Hcy, Hcy + zileuton, or zileuton. (**F**) 5LO DNA methylation levels in the same cells. Data presented are mean ± s.e.m. (*p < 0.05, n = 6 per condition).

To further corroborate the direct involvement of 5LO activation in the Hcy-dependent effect on Aβ metabolism via the effect that Hcy may have on the 5LO promoter methylation status, we treated the N2A APPswe neuronal cells with 50 μM DL-homocysteine for 24 hr in the presence or absence of zileuton (100 μM), a concentration known to significantly block 5LO activation^16^. At the end of the incubation time, supernatants collected and assayed for Aβ 1-40 and LTB4, while cell pellets used to investigate 5LO at the protein and message levels. Compared with vehicle controls, conditioned media from cells incubated with Hcy had a significant elevation of Aβ1-40 levels, which was absent in the presence of zileuton (Fig. 6A). Hcy treatment resulted also in a significant increase in the steady state levels of 5LO proteins, which was not influenced by zileuton (Fig. 6B,C). However, while levels of LTB4 were significantly higher in the Hcy-treated cells than in controls, the presence of zileuton completely prevented this increase (Fig. 6D). On the other hand, quantitative RT-PCR analysis showed that beside the protein also the 5LO mRNA levels were significantly elevated in cells treated with Hcy, whereas 5LO DNA methylation status was significantly decreased and that both of these effects were not influenced by the presence of zileuton (Fig. 6E,F).

## Discussion

In the current paper we provide the first experimental evidence that up-regulation and activation of the 5LO enzymatic pathway is a necessary step for the Hcy-dependent biological effect on the development of the AD-like phenotype in the 3xTg mice. Epidemiological studies have reported the association between high circulating Hcy levels and AD, while longitudinal observations have revealed that the influence of Hcy on AD is independent from other confounders^17^,^18^,^19^,^20^. However, conflicting results have also been published regarding this relationship^21^,^22^.

Since the initial report on this association, studies have suggested several mechanisms that could be responsible for the deleterious effect of high Hcy in the central nervous system including but not limited to oxidative stress, inflammation, and nucleic acids damage^23^,^24^. However, the precise role that Hcy may have with respect to the AD pathogenesis and the relevant mechanisms involved remain to be fully investigated.

In search for novel molecular pathways linking high Hcy with AD, in recent years a methylation hypothesis has been proposed in which Hcy elevation would lead to an increase in SAH, a potent inhibitor of methyl-transfer reactions, and a reduction SAM. The resulting altered ratio in SAM/SAH would then translate in a decreased activity of methyltransferase enzymes which by lowering the methylation state of promoters ultimately would regulate gene expression relevant to the disease pathogenesis^25^,^26^. Among the different genes, we focused our attention on the 5LO whose expression is tightly regulated by DNA methylation and de-methylation of its promoter^27^, and showed that Hcy can indeed modulate 5LO expression level via DNA methylation^12^. Importantly, in the same paper we also demonstrate that the effect of high Hcy on 5LO is a specific one since when we looked at another member of the LO family, the 12/15LO, we did not observe any changes compared with control group^12^. However, whether the 5LO pathway upregulation we observed as result of high Hcy is directly involved and responsible for the modulation of the AD-like phenotype *in vivo* remains to be investigated.

In the current paper first we demonstrated that coincidentally with a significant increase in brain Hcy, SAM levels are reduced and SAH levels increased in the brains of mice receiving the Diet, and that these changes are associated with an up-regulation of the 5LO enzymatic pathway both at the protein and message level. The activation of this pathway was documented by a significant increase in the brain levels of LTB4, the main metabolic product of the enzyme^28^. The biological relevance of the altered SAM/SAH ratio was documented by the significant decrease in the levels of three major methylation enzymes and most importantly by the reduction in the 5LO DNA methylation.

To prove a functional role of 5LO up-regulation in modulating the Hcy effect on the pathological phenotype of 3xTg mice we implemented a pharmacological approach by adding zileuton, a selective and specific inhibitor of 5LO activation, to the Diet. At the end of the study, we observed that 3xTg mice with Diet-induced elevated levels of Hcy manifested a worsening of their memory and learning performances as demonstrated in 3 different paradigms. Thus, compared with controls, these mice had a significant reduction in the percentage of alternation in the Y-maze, which reflects their working memory, but not different with controls in the number of entries, a measure of motor activity. Mice with high Hcy had also signs of impaired memory and learning, as shown in the significant reduction in the cued and contextual recall phases of the fear conditioning paradigm. Similarly, when reference spatial memory function was assessed in the Morris water maze they showed significant impairments compared with controls. By contrast, despite the elevation of brain Hcy and 5LO protein levels, 3xTg treated with zileuton by having the activity of the 5LO blocked did not manifest any of these deficits and their behavioral responses were undistinguishable from the control group.

In accordance with the behavioral tests, we observed that brains from Diet-treated mice had a significant elevation in the amount and deposition of Aβ peptides compared with controls. Confirming previous studies we observed that this increment was mediated by an activation of the γ-secretase pathway, as shown by the significant increase in the steady state levels of 2 major components of the complex, PS1 and APH-1^29^,^30^. However, pharmacological blockade of 5LO activation was sufficient to prevent these changes suggesting an active role of this enzyme in the observed Hcy pro-amyloidotic effect.

Next, we assessed how tau levels and phosphorylation were affected under our experimental conditions. Compared with control, Diet-treated mice had a significant increase in tau phosphorylation at specific epitopes which was associated with an elevation in its insoluble fraction suggesting an effect of Hcy on tau conformation. In an effort to elucidate the potential mechanisms for these changes, we confirmed our previous reports showing that the cdk5 pathway was significantly increased in the Diet-treated group compared with control mice^29^,^30^,^31^. In support of the hypothesis that these changes were driven by the up-regulation of the 5LO enzymatic pathway we observed that 3xTg mice with Diet-induced high Hcy but treated with zileuton did not manifest any alteration in tau phosphorylation or the cdk5 pathway.

To further corroborate the essential role of 5LO in the biological effect of high Hcy on pathways germaine to the development of the AD pathology, and establish that the mechanism involved is Hcy-dependent hypomethylation of 5LO DNA and subsequent up-regulation of this pathway (message, protein and enzymatic activity) we embarked in a series of *in vitro* experiments.

Conditioned media from neuronal cells incubated with Hcy had significant increase in Aβ 1–40 together with a significant elevation in steady state levels of 5LO protein. Importantly, this increase was accompanied by biochemical evidence of an augmented 5LO enzymatic activity as denoted by the significant elevation of LTB4. Confirming our *in vivo* observation in the brains of mice receiving the Diet, we also observed that neuronal cells incubated with Hcy had a significant increase in 5LO mRNA and, most importantly, the methylation of its DNA significantly reduced. Under this experimental condition, while the presence of zileuton blocked the activation of 5LO and the subsequent formation of Aβ 1–40, it had no effect on its protein, mRNA as well as methylation.

In summary, our studies report on a novel biological and functional link between Hcy and the 5LO enzymatic pathway which directly influences both cellular and molecular events germaine to the onset and development of the entire spectrum AD-like phenotype (cognition, amyloid and tau neuropathology). They establish the causative role that the 5LO pathway plays on this biological effect both *in vivo* and *in vitro*, and elucidate a novel mechanism in which high Hcy-dependent 5LO DNA hypomethylation results in 5LO pathway up-regulation, which then is responsible for increased Aβ formation, tau phosphorylation and worsening of behavioral deficits in a relevant mouse model of AD.

The significance of our findings lies in the discovery of a mechanistic interaction between an established environmental risk factor (Hcy) and a genetic risk factor (5LO) in the pathogenesis of AD via epigenetic mechanisms. Taken together, they prove the hypothesis that impaired Hcy metabolism and dysregulation of methylation reactions by modulating gene expression can trigger the activation of an enzymatic pathway which ultimately favors AD onset.

In conclusion, our findings provide novel insights into the molecular mechanisms by which elevated circulating Hcy levels may promote the development of AD-like neuropathology in individuals carrying this environmental risk factor. This biological evidence could ultimately afford us with useful information for novel therapeutic opportunities for preventing or halting AD.

## Materials and Methods

### Animal and treatments

Animal procedures were approved by the Temple University Institutional Animal Care and Usage Committee and in accordance with the Guide for the Care and Use of Laboratory Animals of the National Institute of Health. The 3xTg mice harboring a mutant APP (KM670/671NL), a human mutant PS1 (M146V) knock-in and tau (P301L) transgenes were used in this study^32^. All animals were kept in a pathogen-free environment, on a 12-hour light/dark cycle and had access to food and water ad libitum. The mice were randomized to four groups. Group1 (Control), (n = 5 [2 males and 3 females]), mice were given the standard rodent chow; group 2 (Diet), (n = 6 [3 males and 3 females]) mice were given a standard rodent chow deficient in folate (<0.2 mg/kg), vitamin B6 (<0.1 mg/kg) and B12 (<0.001 mg/kg), which is known to induce HHcy in mice^7^,^9^,^10^; group 3 (Diet plus zileuton), (n = 6 [3 males and 3 females]) mice were given the folate and B vitamins deficient diet and zileuton, and group 4 (zileuton), (n = 5 [3 males and 2 females]) mice were given the standard rodent chow plus zileuton. For the two zileuton groups, the drug was administered in their drinking water at a concentration of 200 mg/L, which we previously showed to be well tolerated and significantly suppress 5LO enzymatic activity^16^. Considering that each mouse drinks in average 3–4 ml/day of water, the final concentration of the active drug was approximately 0.6–0.8 mg/day.

Starting at 5 months of age, mice were randomized to the four groups and followed for 7 months until they were 12 month-old. At this age time-point, they underwent behavioral testing and then euthanized. During the study, mice in all four groups gained weight regularly, and no significant differences in weight were detected among them by the end of the study (data not shown). No macroscopic effect on the overall general health was observed in any of the four groups. No macroscopic differences among the four groups were observed when major organs such as liver, spleen, heart and kidneys we compared at sacrifice. After euthanasia, animals were perfused with ice-cold 0.9% Phosphate Buffered saline (PBS) containing 10 mM EDTA, pH 7.4. Brain was removed and dissected in two halves by mid-sagittal dissection. One half was immediately stored at −80 °C for biochemistry assays, the other immediately immersed in 4% paraformaldehyde overnight for immunohistochemistry studies.

### Behavioral Tests

All animals were pre-handled for 3 days prior testing, they were tested in a randomized order, and all tests conducted by an experimenter blinded to the treatments.

### Y-maze

The Y-maze apparatus consisted of three arms 32 cm (long) 610 cm (wide) with 26-cm walls (San Diego Instruments, San Diego, CA). Testing was always performed in the same room and at the same time to ensure environmental consistency as previously described^29^,^33^. Briefly, each mouse was placed in the center of the Y-maze and allowed to explore freely through the maze during a 5-min session. The sequence and total number of arms entered were video recorded. An entry into an arm was considered valid if all four paws entered the arm. An alternation was defined as three consecutive entries in three different arms (i.e. 1, 2, 3 or 2, 3, 1, etc). The percentage alternation score was calculated using the following formula: Total alternation number/ (total number of entries-2)*100.

### Fear-conditioning

The fear conditioning test paradigm was performed following methods previously described^29^,^33^. Briefly, the test was conducted in a conditioning chamber (19 × 25 × 19 cm) equipped with black methacrylate walls, transparent front door, a speaker and grid floor (Start Fear System; Harvard Apparatus). On day one, mice were placed into the conditioning chamber and allowed free exploration for 2 min in the white noise (65 Db) before the delivery of the conditioned stimulus (CS) tone (30 s, 90 Db, 2000 Hz) paired with a foot-shock unconditioned stimulus (US; 2 s, 0.6 mA) through a grid floor at the end of the tone. A total of 3 pairs of CS-US pairing with a 30 s inter trial interval (ITI) were presented to each animal in the training stage. The mouse was removed from the chamber 1 min after the last foot-shock and placed back in its home cage. The contextual fear conditioning stage started 24 h after the training phase when the animal was put back inside the conditioning chamber for 5 min with white noise only (65 dB). The animal’s freezing responses to the environmental context were recorded. The tone fear conditioning stage started 2 h after the contextual stage. The animal was placed back to the same chamber with different contextual cues, including white wall, smooth metal floor, lemon extract drops, and red light condition. After 3 min of free exploration, the mouse was exposed to the exactly same 3 CS tones with 30 s ITI in the training stage without the foot-shock and its freezing responses to the tones were recorded.

### Morris Water Maze

The Morris Water Maze test was performed with the ANY-maze System (Stoelting Co. Wood Dale, IL) as previously described^29^,^33^. Briefly, the apparatus used was a white circular plastic tank (122 cm in diameter) with walls 76 cm high, filled with water maintained at room temperature, which was made opaque by the addition of a nontoxic white paint, and inside had a removable, square (10 cm in side length) plexiglass platform. The tank was located in a test room containing various prominent visual cues. Mice were trained to swim to the platform submerged 1.5 cm beneath the surface of the water and invisible to the mice while swimming. The platform was located in a fixed position, equidistant from the center and the wall of the tank. Mice were subjected to four training trials per day (inter-trial interval, 15 minutes). During each trial, mice were placed into the tank at one of four designated start points in a random order. Mice were allowed to find and escape onto the submerged platform. If they failed to find the platform within 60 seconds, they were manually guided to the platform and allowed to remain there for 10 seconds. Mice were trained to reach the training criterion of 20 seconds (escape latency). They were assessed in the probe trial 24 hours after the last training session and consisted in a 60-second free swim in the pool without the platform. Each animal’s performance was recorded for the acquisition parameters (latency to find the platform) and the probe-trial parameters (number of entries to the platform and time in quadrants).

### Cells and treatment

Neuro-2 A neuroblastoma (N2A) cells stably expressing human APP carrying the K670 N, M671L Swedish mutation (N2A-APPswe) were cultured in Dulbecco’s modified Eagle medium supplemented with 10% fetal bovine serum, 100 U/mL streptomycin (Cellgro, Herdon, VA) and 400 mg/mL G418 (Invitrogen, Carlsbad, CA) at 37 °C in the presence of 5% CO2. The cells were cultured to 80% to 90% confluence in six-well plates as previously described^12^,^14^. Briefly, at this point medium was changed with fresh medium containing 50 μM DL-homocysteine (Sigma, St Louis, MO) with 40 μM adenosine (Sigma, St Louis, MO) and 10 μM erythro-9-(2-hydroxy-3-nonyl)-adenine hydrochloride (EHNA) (Sigma, St Louis, MO) as previously described^11^,^29^, in the presence of zileuton or its vehicle (control). After 24 hrs treatment, media were collected for biochemical measurement, and cell harvested for Western blotting analyses and quantitative real time RT-PCR.

### Biochemical analyses

Brain tissues were homogenized and sequentially extracted in RIPA and then formic acid (FA), where the RIPA fraction contains the soluble, whereas the FA fraction the insoluble forms of the Aβ peptides and tau protein, as previously described^29^,^33^. Aβ1-40 and Aβ1-42 levels were assayed by a sensitive sandwich ELISA kits (WAKO Chem., Richmond, VA). Analyses were always performed in duplicate and in a coded fashion. For the analysis of Hcy, SAH and SAM levels in the brain, tissue aliquots were frozen in liquid nitrogen and reduced into a fine powder using mortar and pestle. The powder was re-suspended in 100 μl of NKPD buffer (2.68 mM KCl, 1.47 mM KH2PO4, 51.10 mM Na2HPO4, 7.43 mM NaH2PO4, 62 mM NaCl, 1 mM EDTA, and 1 mM dithiothreitol) and sonicated at 40 watts and 70% duty cycle for about 2 min, then clarified by centrifugation at 10,000x g for 15 min. Measurements were performed by HPLC analysis using Waters AccQ.Fluor derivitizing reagents (Waters Corp., MA) as previously described^34^,^35^. The final concentration of Hcy, SAH or SAM in the samples was always normalized by mg protein. Analyses were always performed in triplicate and in a coded manner.

### Western Blot Analyses

RIPA fractions of brain homogenates or cell lysates were used for western blot analyses as previously described^29^,^33^. Briefly, samples were electrophoresed on 10% Bis–Tris gels or 3–8% Tris–acetate gel (Bio-Rad, Richmond, CA), transferred onto nitrocellulose membranes (Bio-Rad, Richmond, CA), and then incubated overnight with the appropriate primary antibodies as indicated in Table 2. After three washings with T-TBS (pH7.4), membranes were incubated with IRDye 800CW-labeled secondary antibodies (LI-COR Bioscience, Lincoln, NE) at RT for 1 h. Signals were developed with Odyssey Infrared Imaging Systems (LI-COR Bioscience, Lincoln, NE). β-actin was always used as internal loading control.

### Immunohistochemistry

Immunostaining was performed as described in our previous studies^29^,^33^. Briefly, serial coronal sections were mounted on 3-aminopropyl triethoxysilane (APES)-coated slides. Every eighth section from the habenular to the posterior commissure (6–8 sections per animal) was examined using unbiased stereological principles. The sections for testing Aβ were deparaffinized, hydrated, pretreated with formic acid (88%) and subsequently with 3% H_2_O_2_ in methanol. The sections used for testing HT7, AT8, AT180, AT270, synaptophysin, PSD95 and MAP2 were deparaffinized, hydrated and subsequently treated with 3% H_2_O_2_ in methanol, and then antigen retrieved with 10 mM sodium citrate buffer. Sections were blocked in 2% fetal bovine serum before incubation with the appropriate primary antibody overnight at 4 °C. After washing, sections were incubated with biotinylated anti-mouse IgG (Vector Lab, Burlingame, CA) and then developed by using the avidin-biotin complex method (Vector Lab, Burlingame, CA) with 3,3′-diaminobenzidine (DAB) as a chromogen. Light microscopic images were captured using software QCapture 2.9.13 (Quantitation Imaging Corporation, Surrey, Canada) with the auto-exposure option. These images were used to calculate the area occupied the immunoreactivities using the software Image-ProPlus (Media Cybernetics).

### Quantitative Real Time RT-PCR

RNA was extracted and purified using the RNeasy mini-kit (Qiagen, Valencia, CA), as previously described^36^. Briefly, 1 μg of total RNA was used to synthesize cDNA in a 20 μl reaction using the RT2 First Strand Kit for RT-PCR (SuperArray Bioscience, Frederick, MD). 5LO genes were amplified by using the proper primers obtained from Super-Array Bioscience. β-Actin was used as an internal control gene to normalize for the amount of RNA. Quantitative real-time RT-PCR (qRT-PCR) was performed by using StepOnePlus Real-Time PCR Systems (Applied Biosystems, Foster City, CA). Two microliters of cDNA was added to 10 μl of SYBR Green PCR Master Mix (Applied Biosystems, Foster City, CA). Each sample was run in duplicate, and analysis of relative gene expression was done by StepOne software v2.1 (Applied Biosystems, Foster City, CA).

### DNA Methylation assay

To measure the 5-LO DNA methylation levels, we used the restriction digest-qPCR assay as previously described^12^. The method utilizes the ability of methylation-sensitive endonucleases to digest only un-methylated recognition sites, and their inability to act on sites with methylated cytosine. Thus, if the targeted ∗CpG is methylated, the site is blocked for the enzyme’s endonuclease activity and as a result; greater amounts of templates are available for the action of the Taq DNA polymerase^37^. Briefly, genomic DNA was extracted from the mouse brain cortex or N2A-APPswe cells using PureLink Genomic DNA Mini kit (Invitrogen, Carlsbad, CA) according to the manufacturer’s protocol. 1 μg of genomic DNA was used in a restriction digest reaction for two methylation-sensitive endonucleases, BstUI and HpaII. Digested DNA samples were diluted with water and an aliquot (100 ng DNA) was used for qPCR with SYBR Green PCR Master Mix (Applied Biosystems, Foster City, CA) as described in the method of quantitative real time RT-PCR.

### Data Analysis

Data analyses were performed using SigmaStat for Windows version 3.00. Statistical comparisons were performed by Unpaired Student’s t-test or the Mann-Whitney rank sum test when a normal distribution could not be assumed. Values in all figures and table represent mean ± S.E.M. Significance was set at p < 0.05.

## Additional Information

**How to cite this article:** Li, J.-G. *et al*. Five lipoxygenase hypomethylation mediates the homocysteine effect on Alzheimer’s phenotype. *Sci. Rep.* **7**, 46002; doi: 10.1038/srep46002 (2017).

**Publisher's note:** Springer Nature remains neutral with regard to jurisdictional claims in published maps and institutional affiliations.

## Figures and Tables

**Table 1:** Levels of Hcy, SAM, SAH and SAH/SAM ratios in brain homogenates from 3xTg mice receiving chow diet (control), Folate and B vitamins deficient diet (Diet), Diet plus zileuton, or zileuton starting at 5 months of age until 12-month-old.

	3xTg Control	3xTg Diet	3xTg Diet + zileuton	3xTg zileuton	P
Hcy (pg/mg tissue)	289 ± 4	351 ± 12*	337 ± 7*	285 ± 5	*< 0.05
SAM (mmol/g)	14 ± 1	9.7 ± 1*	10 ± 0.8*	14.6 ± 1	*00.1
SAH (mmol/g)	6.6 ± 0.8	12.2 ± 1.6*	10 ± 0.6*	6 ± 0.7	*0.0006
SAH/SAM	0.47	1.2	1	0.41	

**Table 2:** Antibodies used in the study.

Antibody	Immunogen	Host	Application	Source	Catalog Number
4G8	aa 18-22 of human beta amyloid (VFFAE)	Mouse	IHC	Covance	SIG-39220
APP	aa 66–81 of APP {N-terminus}	Mouse	WB	Millipore	MAB348
BACE-1	aa human BACE (CLRQQHDDFADDISLLK)	Rabbit	WB	IBL	18711
ADAM10	aa 732–748 of human ADAM 10	Rabbit	WB	Millipore	AB19026
PS-1	aa around valine 293 of human presenilin 1	Rabbit	WB	Cell Signaling	3622S
Nicastrin	aa carboxy-terminus of human Nicastrin	Rabbit	WB	Cell Signaling	3632
APH-1	Synthetic peptide from hAPH-1a	Rabbit	WB	Millipore	AB9214
Pen-2	aa N-terminal of human and mouse Pen-2	Rabbit	WB	Invitrogen	36–7100
HT-7	aa 159–163 of human tau	Mouse	WB, IHC	Thermo	MN1000
AT-8	Peptide containing phospho-S202/T205	Mouse	WB, IHC	Thermo	MN1020
AT-180	Peptide containing phospho-T231/S235	Mouse	WB, IHC	Thermo	P10636
AT-270	Peptide containing phospho-T181	Mouse	WB, IHC	Thermo	MN1050
PHF-13	Peptide containing phospho-Ser396	Mouse	WB, IHC	Cell Signaling	9632
PHF-1	Peptide containing phospho-Ser396/S404	Mouse	WB, IHC	Dr. P. Davies	Gift
PSD95	Purified recombinant rat PSD-95	Mouse	WB, IHC	Thermo	MA1-045
SYP	aa 221–313 of SYP of human origin	Mouse	WB, IHC	Santa Cruz	sc-55507
MAP2	Purified Microtubule-associated protein from rat brain	Rabbit	WB, IHC	Millipore	AB5622
GFAP	spinal chord homogenate of bovine origin	Mouse	WB	Santa Cruz	sc-33673
CD45	aa 1075–1304 of CD45 of human origin	Rabbit	WB	Santa Cruz	sc-25590
DNMT1	aa 1317–1616 mapping near the C-terminus of Dnmt1 of human origin	Rabbit	WB	Santa Cruz	sc-20701
DNMT3α	aa 1–295 of Dnmt3α of human origin	Rabbit	WB	Santa Cruz	sc-20703
DNMT3β	aa 1–230 mapping near the N-terminus of Dnmt3β of human origin	Rabbit	WB	Santa Cruz	sc-20704
Actin	gizzard Actin of avian origin	Mouse	WB	Santa Cruz	sc-47778

WB: Western blot; IHC: immunohistochemistry.
